# Analysis of oxygen vacancy in Co-doped ZnO using the electron density distribution obtained using MEM

**DOI:** 10.1186/s11671-015-0887-2

**Published:** 2015-04-18

**Authors:** Ji Hun Park, Yeong Ju Lee, Jong-Seong Bae, Bum-Su Kim, Yong Chan Cho, Chikako Moriyoshi, Yoshihiro Kuroiwa, Seunghun Lee, Se-Young Jeong

**Affiliations:** Department of Cogno-Mechatronics Engineering, Pusan National University, Miryang, 627-706 South Korea; Department of Nanofusion Engineering, Pusan National University, Busan, 609-735 South Korea; Busan Center, Korea Basic Science Institute, Busan, 618-230 South Korea; Frontier in Extreme Physics, Korea Research Institute of Standards and Science, Daejeon, 305-340 South Korea; Department of Physical Science, Hiroshima University, Higashi-Hiroshima, 739-8526 Japan; The Institute of Basic Science, Korea University, Seoul, 136-713 Republic of Korea; Current address: Department of Materials Science and Engineering, University of Maryland, College Park, MD 20742 USA

**Keywords:** ZnO, Rietveld refinement, Maximum entropy method, Oxygen vacancy, Co-doped ZnO

## Abstract

Oxygen vacancy (V_O_) strongly affects the properties of oxides. In this study, we used X-ray diffraction (XRD) to study changes in the V_O_ concentration as a function of the Co-doping level of ZnO. Rietveld refinement yielded a different result from that determined via X-ray photoelectron spectroscopy (XPS), but additional maximum entropy method (MEM) analysis led it to compensate for the difference. V_O_ tended to gradually decrease with increased Co doping, and ferromagnetic behavior was not observed regardless of the Co-doping concentration. MEM analysis demonstrated that reliable information related to the defects in the ZnO-based system can be obtained using X-ray diffraction alone.

## Background

Oxygen vacancy (V_O_), one of the representative native defects in oxides, has received much attention because of the important role played thereby in determining the physical properties of materials [1-3]. Various tools have been used for the qualitative and quantitative analysis of V_O_. These include photoluminescence [4], ultraviolet-visible (UV-vis) [5], Raman [5], and X-ray photoelectron spectroscopic (XPS) techniques [6]. Quantitative analyses of V_O_ have been successfully performed via Rutherford backscattering spectroscopy [7] or X-ray absorption spectroscopy using synchrotron radiation [8]. Additionally, X-ray diffraction (XRD) is a simple and useful tool for analysis of V_O_ because it reveals the crystal structure and the electron density distribution of periodic arrays of atoms [9].

Fitting of X-ray diffraction data using the Rietveld refinement has been attempted for the quantitative analyses of V_O_ in terms of oxygen site occupancy [10,11]. However, additional corrections and the use of neutron or synchrotron X-rays were required [11-13]. Electron density profiling using the maximum entropy method (MEM) is also a suitable tool for analysis of V_O_ because it uses the more precise Rietveld refinement that resolves summation-terminated errors and affords a better structural model [14,15]. Furthermore, MEM introduces negligible modeling errors via least-biased electronic reconstruction of X-ray diffraction patterns in real space [15].

We sought to confirm whether MEM analysis could be used for analysis of V_O_. In this study, we applied such analysis to V_O_ that changed as a function of the Co-doping concentration in ZnO. Co-doped ZnO is a good candidate room-temperature magnetic semiconductor and has been intensively studied in terms of intrinsic ferromagnetism. However, the origin of such ferromagnetism remains controversial, and the presence thereof limits applications of the semiconductor. V_O_ was regarded, until recently, to explain the ferromagnetism and was reported to be affected by Co-doping concentration [8]. Herein, we analyzed the change in V_O_ as a function of Co-doping concentration and compared the results with XPS data. A method of analysis of V_O_ is proposed, using conventional XRD and MEM techniques.

## Methods

ZnO and Co-doped ZnO (Zn_1−x_Co_x_O, *x* = 0.01, 0.05, 0.1) powder samples were fabricated by sol-gel methods [16,17]. Zinc acetate dihydrate (Sigma-Aldrich, St. Louis, MO, USA) and cobalt acetate tetrahydrate (Sigma-Aldrich, St. Louis, MO, USA), used as starting materials, were dissolved in 2-methoxyethanol (Sigma-Aldrich, St. Louis, MO, USA) and stabilized by monoethanolamine (Sigma-Aldrich, St. Louis, MO, USA). To exclude the possibility of external contamination, the dissolution and drying processes were performed under a pure argon gas (99.999% purity) atmosphere, and under vacuum, each for 10 h, respectively. The organic residuals in samples were completely removed via an intermediate heat treatment at 300°C and a subsequent final heat treatment at 800°C under vacuum for 10 h [16]. The samples used in this study were characterized using XRD, and we found a high degree of crystallinity, which was comparable to that of commercially available high-quality powder samples (ZnO; CAS 1314-13-2, Sigma-Aldrich, St. Louis, MO, USA). We also characterized the samples using synchrotron radiation, and we found high sample quality. A characterization study using synchrotron radiation will be submitted to a specialized journal soon. XRD (Empyrean Series 2, PANalytical) experiments were performed to analyze the crystal structures and electron density distributions of the powder samples. The Rietveld and MEM analyses were performed using a published technique [18]. The MEM calculation was performed using ENIGMA software [19] with 66 × 66 × 104 pixels. The electron density distribution was reconstructed using the VESTA visualization program [20]. An X-ray photoelectron spectrometer (model: Theta Probe (Thermo Electron Co., Waltham, MA, USA), Korean Basic Science Institute, Busan Center) was used for atomic composition analysis. Magnetic-field-dependent magnetization was measured using a vibrating sample magnetometer (VSM) equipped with a physical property measurement system (PPMS; Model 6000, Quantum Design, San Diego, CA, USA).

## Results and discussion

**Figure 1:**
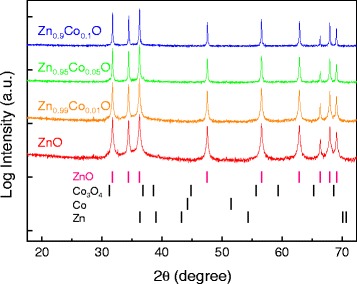
XRD patterns of ZnO and Zn_1−x_Co_x_O (*x* = 0.01, 0.05, 0.1) with Bragg peak positions for ZnO, Co_3_O_4_, Co, and Zn metal.

Figure 1 shows the XRD patterns of the ZnO and ZnCoO powder samples. The Bragg peaks correspond to the wurtzite ZnO structure and other possible secondary species [16,21]. The diffraction intensity of each sample is presented logarithmically to better observe the background levels. Figure 1 shows that the XRD patterns of all samples had pronounced diffraction peaks that corresponded to wurtzite ZnO, with no additional peaks corresponding to secondary phases (at least within the detection limits). This indicates that the Co^2+^ ions were well-substituted during doping into Zn sites, without creation of secondary species [16,22]. Doping Co into ZnO tended to decrease the full-width at half-maximum (FWHM) values of XRD peaks. To quantitatively analyze this trend, the grain sizes of each powder sample were calculated using the Scherrer equation [23]. These sizes were estimated to be 93, 107, 115, and 143 nm for ZnO, Zn_0.99_Co_0.01_O, Zn_0.95_Co_0.05_O, and Zn_0.9_Co_0.1_O, respectively. Co doping thus improved the crystallinity of the samples, in proportional to the Co-doping concentration, in our experimental range [24]. The crystallinity of ZnO can be greatly affected by lattice strain [25] or native defects such as V_O_ [26-28] and interstitial zinc (Zn_i_) [27,28]. The contribution of lattice strain (resulting from the Co-doping concentration) to differences in crystallinity was assumed to be negligible because all powder samples were post-annealed at both 300°C and 800°C [17,29], which would relieve any lattice strain. Co doping was thought to induce insignificant amounts of lattice strain that might result from a difference in ionic radii, because the ionic radius of Zn^2+^ (74 pm) is similar to that of Co^2+^ (72 pm) [30]. Consequently, the observed enhancement in the crystallinity of the samples was attributed to a decrease in the density of crystal defects caused by Co doping. A quantitative analysis of V_O_ and Zn_i_ was performed to explore this hypothesis; we refined the XRD patterns of the samples.

**Figure 2:**
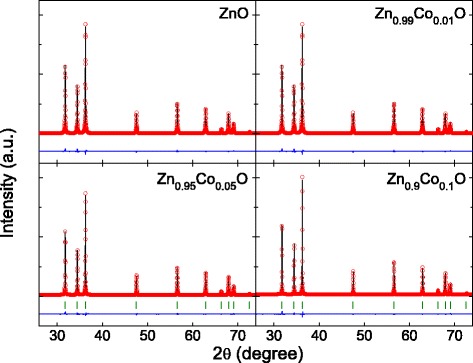
Powder XRD patterns and Rietveld refinement results. Powder XRD patterns (black lines) and Rietveld refinement results (red circles) of ZnO, Zn_0.99_Co_0.01_O, Zn_0.95_Co_0.05_O, and Zn_0.9_Co_0.1_O samples. The differences between the XRD patterns and Rietveld refinement results are indicated by the lower blue lines. The green bars provide the calculated Bragg peak positions.

Figure 2 shows the Rietveld refinement results for the ZnO and ZnCoO samples performed using single-phase wurtzite ZnO. The reliability factors based on the Bragg intensity (*R*_*I*_) and structure factor (*R*_*F*_) were below 1.5% and 0.8%, respectively, for all samples (Table 1). The refinement results revealed that the lattice constants steadily increased with increasing Co-doping level. It is known that formation of Zn_i_ leads to an increase in lattice constants [31], while formation of V_O_ induces a decrease in lattice constants [32]. The observed reduction of internal defects, and the increased lattice constants with increasing Co-doping level, indicated that the main defects embedded in the ZnO sample were V_O_, and hence, their density decreased as the Co-doping concentration increased.

**Table 1:** **Reliability factors of ZnO and ZnCoO samples**

**Sample**	***R***_***I***_ **(%)**	***R***_***F***_ **(%)**	***a*** **(Å)**	Δ***g***_***O***_ **(%)**
ZnO	1.473	0.731	3.24989	-
Zn_0.99_Co_0.01_O	1.066	0.605	3.25084	−0.39
Zn_0.95_Co_0.05_O	0.942	0.509	3.25120	2.03
Zn_0.9_Co_0.1_O	0.776	0.414	3.25235	3.48

Reliability factors based on the Bragg intensity (*R*_*I*_) and structure factor (*R*_*F*_), lattice constants (*a*), and relative change in oxygen site occupancies (Δ*g*_*O*_) of ZnO and ZnCoO samples. *g*_*O*_ refers to the site occupancy at the oxygen atom in the Rietveld refinement.

An additional Rietveld refinement of the oxygen occupancies in each finalized Rietveld refinement was performed to examine the validity of this concept. Table 1 also lists the change in the oxygen occupancy [Δ*g*_*O*_ = (*g*_*O*_(ZnO) − *g*_*O*_(ZnCoO))/*g*_*O*_(ZnO)] for each sample, where *g*_*O*_ refers to oxygen site occupancy. The oxygen occupancy increased for Zn_0.99_Co_0.01_O (i.e., negative Δ*g*_*O*_ was obtained) but decreased for Zn_0.95_Co_0.05_O and Zn_0.9_Co_0.1_O. Also, the oxygen vacancy was greater for Zn_0.95_Co_0.05_O than for Zn_0.9_Co_0.1_O. MEM analysis was used to further examine this trend.

**Figure 3:**
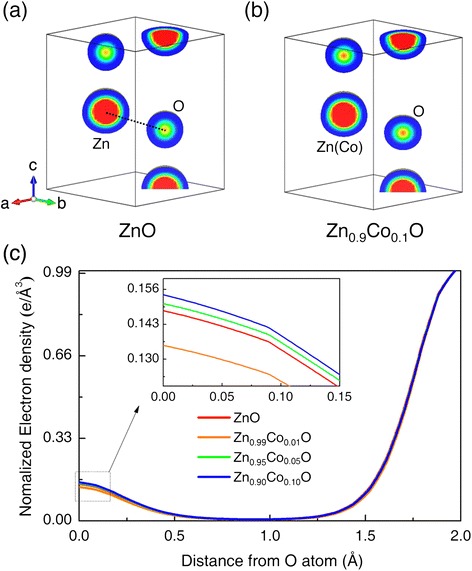
Electron density distribution and line profiles. Electron density distribution of **(a)** ZnO and **(b)** Zn_0.9_Co_0.1_O on the (110) plane obtained from Rietveld/MEM analyses. **(c)** Electron density line profiles of the ZnO and ZnCoO samples along the O-Zn(Co) bond (indicated by the dotted line in **(a)**). The inset is a magnification of the line profiles around the O atoms.

It was expected that the presence of V_O_ in ZnO and ZnCoO would induce changes in the electron density distributions of the oxygen atoms. Hence, the electron densities of the oxygen atoms as a function of Co-doping concentration were investigated via MEM/Rietveld analysis [18]. Figure 3a,b show electron density maps of the ZnO and Zn_0.9_Co_0.1_O samples in the (110) plane, respectively. The Zn sites of the ZnO sample and the Zn(Co) sites of the Zn_0.9_Co_0.1_O sample did not noticeably differ in electron density. This is because of the low-dopant concentration used, and because Zn and Co exhibited similar electron density distributions, as Zn^2+^ and Co^2+^ have similar total numbers of electrons [33,34]. However, the electron densities at the central O atoms of ZnO and Zn_0.9_Co_0.1_O were clearly different, thus, 20.65 and 21.91 e/A^3^, respectively. This indicated that the oxygen sites in the wurtzite ZnO structure became increasingly occupied by oxygen atoms after Co doping. Thus, Co doping decreased the V_O_ content.

Figure 3c shows the electron density line profiles along the direction of the O-Zn bond. These profiles enable precise analysis of oxygen occupancy as a function of the Co content. The lines were normalized to the electron density at the Zn(Co) atomic position to allow comparison of V_O_ with Zn occupancy. The electron density at the O atomic position increased in the order Zn_0.99_Co_0.01_O < ZnO < Zn_0.95_Co_0.05_O < Zn_0.9_Co_0.1_O, in agreement with the Rietveld refinement results. The sample with 1% Co doping exhibited significantly lower oxygen electron density, which did not agree with the Rietveld refinement data.

**Figure 4:**
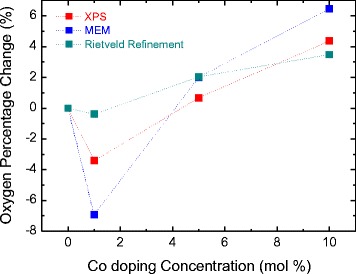
Change in oxygen occupancy (∆*g*_*O*_) analyzed from the Rietveld refinement, MEM, and XPS studies.

XPS was used to rationalize the conflicting V_O_ results. Figure 4 shows the percentage change in oxygen occupancy as a function of Co concentration as determined by the Rietveld refinement, MEM, and XPS techniques. The MEM data were calculated from the integrals of the electron density profiles of the Zn(Co) and O atom positions shown in Figure 3c and the ratio of the oxygen electron density area to the Zn(Co) electron density area. The change in oxygen occupancy revealed by XPS was obtained from the integrals of the areas of the Zn 2p, Co 2p, and the O 1s peaks. The calculation of change in oxygen occupancy was performed on the basis of a pure ZnO sample without Co dopant, as per the data of Table 1. The MEM and XPS results both showed that the sample with 1 mol% Co doping exhibited an abrupt drop in oxygen content; the samples with more doping had increased oxygen contents, in agreement with both the Rietveld refinement and the MEM results. Consequently, the XPS profile results were in better agreement with the MEM results than with the data from the Rietveld refinement. This indicated that MEM analysis, via repeated error correction, provided more accurate structural information. These findings suggest that reliable defect analysis may be possible using easily accessed laboratory X-ray data.

**Figure 5:**
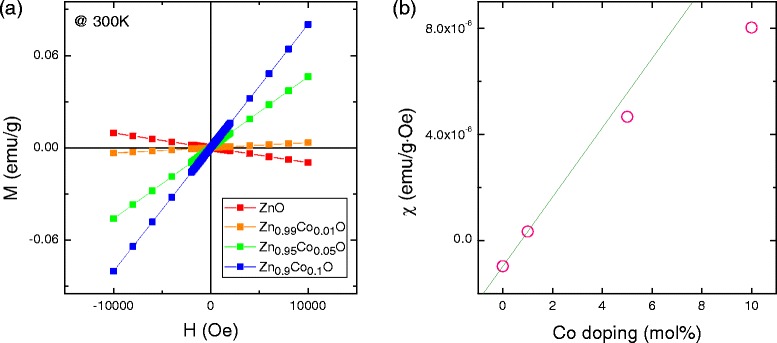
M-H curves and magnetic susceptibilities. **(a)** M-H curves of ZnO with different Co-doping levels. **(b)** Magnetic susceptibilities as a function of Co doping.

The magnetic field dependences of magnetization (M-H curves) were measured for all samples (Figure 5a). Pure ZnO is diamagnetic, and Co-doped ZnO exhibits paramagnetic behavior because of the 3d electron of Co^2+^. We reconfirmed that the ZnCoO samples were not intrinsically ferromagnetic, regardless of Co concentration [6,16]. Figure 5b shows the magnetic susceptibilities, which are the slopes of the M-H curves; these increased nonlinearly with increased Co-doping level. With increasing Co concentration, not all Co spins behave paramagnetically; some spins assume configurations differing in alignment. Considering the absence of secondary phases in the above structural analysis, we conjecture that increasing numbers of Co atoms assuming positions neighboring oxygen atoms created an antiferromagnetic configuration via superexchange interaction.

The observed trend, that creation of V_O_ was suppressed with increasing Co-doping level, is attributable to differences in the Zn-O and Co-O bond strengths; the O^2−^ ions in the wurtzite ZnO structure are tetrahedrally coordinated and thereby form four Zn-O bonds [35]. Doping of Co^2+^ ions into ZnO creates Co-O bonds, the diatomic bond dissociation energy of which is higher than that of the Zn-O bond by 84 kJ/mol (Zn-O: 284 kJ/mol, Co-O: 368 kJ/mol) [36]. This indicates that the Co-O bonds created by Co doping enhanced the average bond strength between oxygen ions and neighboring cations, i.e., doping decreased the possibility of oxygen-cation bond dissociation during sample fabrication or post-treatment processing [36]. The experimental results indicate that V_O_ decreased at high-level Co doping (i.e., above 5 mol%). The supporting analyses suggest that Co doping can impede creation of V_O_. However, the abrupt increase of V_O_ at 1 mol% of Co doping is not well-understood and warrants additional study.

## Conclusions

The Rietveld refinement results of the X-ray diffraction patterns of the ZnCoO system indicated that increased Co doping of ZnO tended to decrease the V_O_, but the V_O_ increased slightly upon 1 mol% of Co doping. The MEM results were in better agreement with the XPS data, which indicated that MEM analysis could be a reliable tool for the study of V_O_. Additional research is needed to explain the anomalous behavior at 1 mol% of Co doping. More advanced X-ray electron density studies using synchrotron radiation would provide more precise and reliable data, but nevertheless, our present work shows that MEM is a reliable technique for the analysis of defects in materials characterized by XRD, which is a readily accessible tool in the material scientist laboratory. This approach will be of particular value in early-stage studies of oxide systems.
